# Supplementary material for: Response and inversion of skewness parameters to meteorological factors based on RGB model of leaf color digital image
Source: PLoS One. 2023 Nov 15;18(11):e0288818. doi: 10.1371/journal.pone.0288818 (PMC10650994; doi:10.1371/journal.pone.0288818)
Supplement: S1 File — (DOC) [file pone.0288818.s001.doc]

***Supplementary Material***


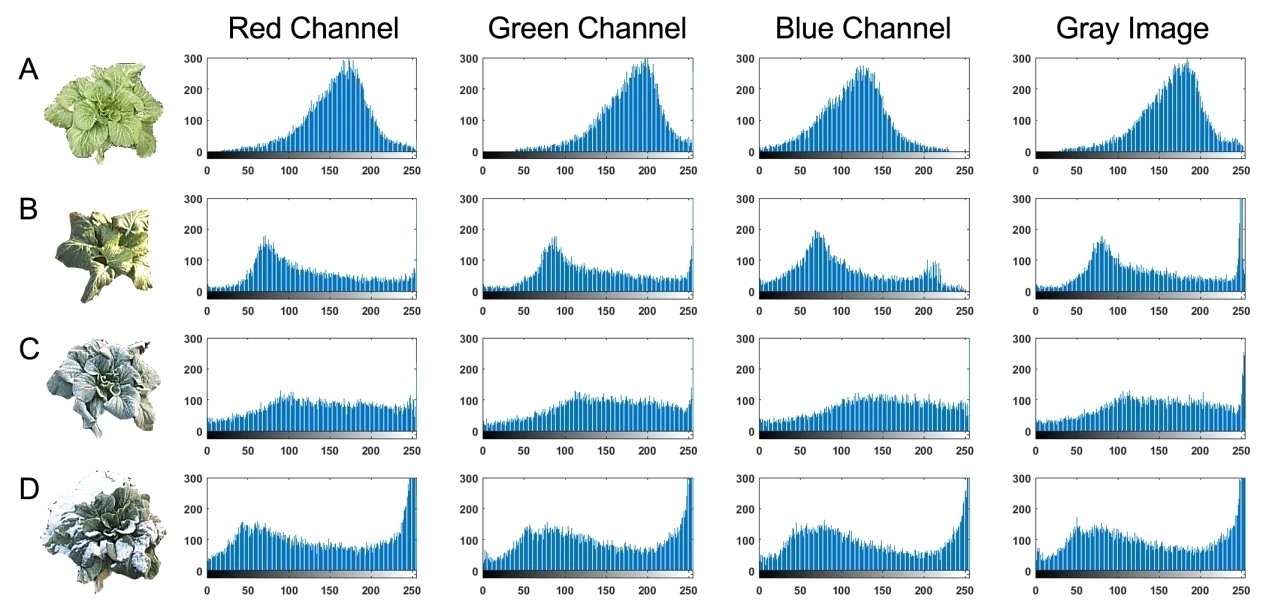


**S1 Fig.** RGB model color gradation distribution of four types of pakchoi canopy images. Four types of pakchoi canopy were selected to represent the image types: normal light (A), direct sunlight (B), frost cover (C), and snow cover (D). The cumulative frequency histogram of red, green and blue channels, as well as gray-level images, were drawn using the imhist function of MATLAB. The X-axis is the cumulative frequency, and the Y-axis is the intensity level frequency.


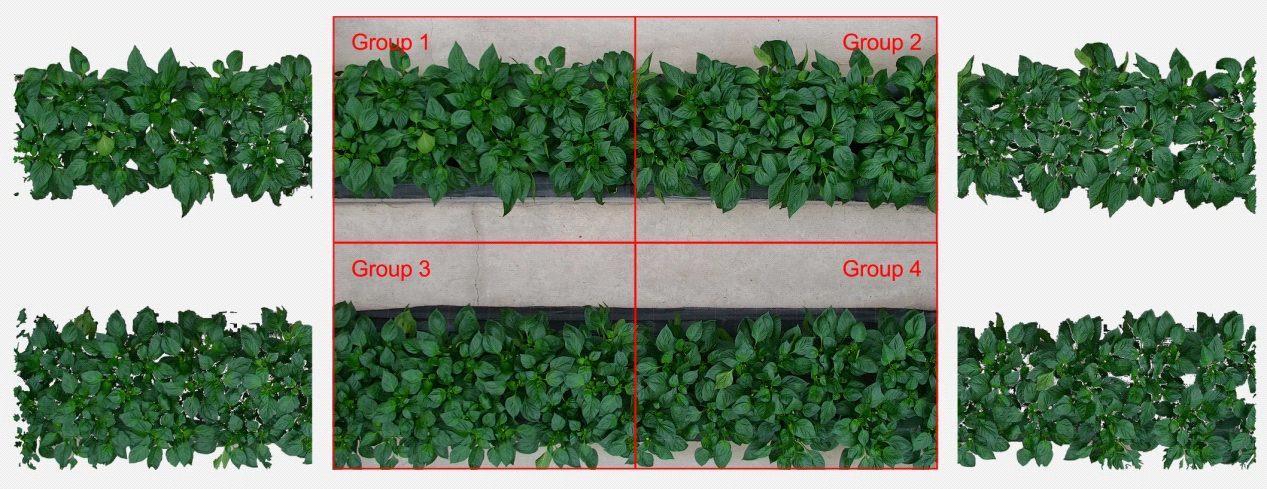


**S2 Fig**. Image grouping scheme and image separation effect of pepper in greenhouse


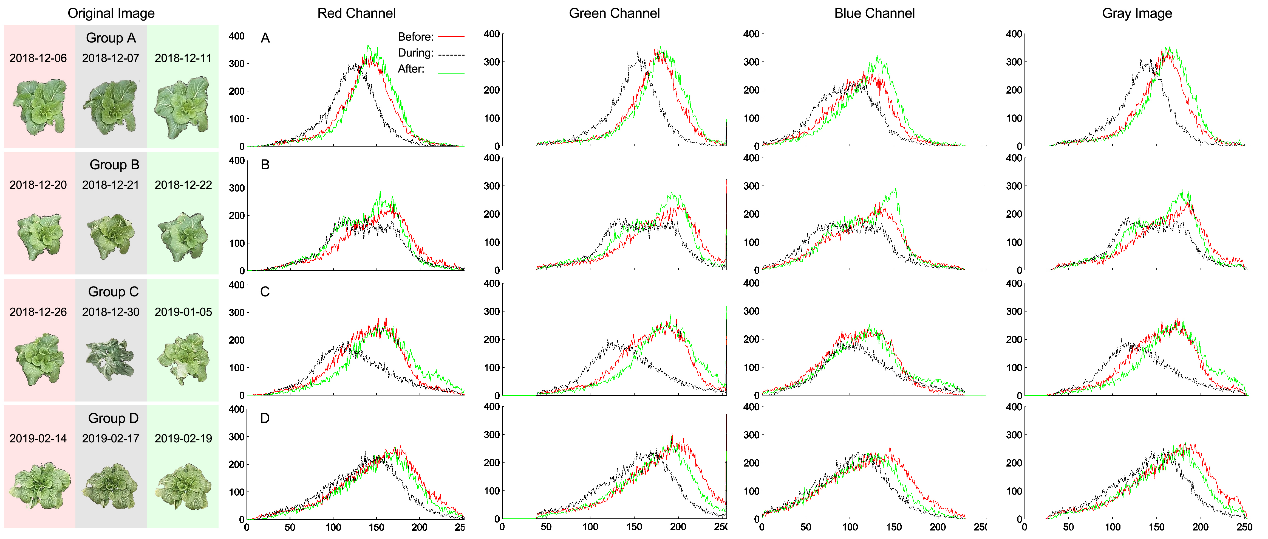


**S3 Fig.** The cumulative frequency broken line graph of color gradation distribution of red, green and blue channels, as well as gray-level images of pakchoi canopy images of four cooling processes. Four cooling processes were selected: Group A-D. In the figure, the original image background of pakchoi canopy before, during, and after cooling processes are denoted as red, gray, and green. The *Plot* function of MATLAB software was used to draw the cumulative frequency broken line graph of color gradation distribution of red, green and blue, as well as gray-level images of left images: before cooling process (Before), during cooling process(During) and after cooling process(After). The X-axis is the cumulative frequency, and the Y-axis is the intensity level frequency.

| Normal distribution test | Red channel | | Green channel | | Blue channel | | Gray-level image | |
| --- | --- | --- | --- | --- | --- | --- | --- | --- |
|  | *H* | *P* | *H* | *P* | *H* | *P* | *H* | *P* |
| Lilliefors test | 1 | 0.001 | 1 | 0.001 | 1 | 0.001 | 1 | 0.001 |
| Jarque-Bera test | 1 | 0.001 | 1 | 0.001 | 1 | 0.001 | 1 | 0.001 |

**S1 Table.** Normal test of color distribution of pakchoi canopy at 08 AM. The *Lillietest* and *JBtest* functions of MATLAB software were used to carry out Lilliefors Test and JarqueBera Test on the distribution of the double precision array of red, green and blue channels, as well as gray-level images of the canopy color images. The return values of H were all 1, indicating that the gradation distribution of canopy color did not accord with normal distribution. The P values of the occurrence probability of the event were less than 0.05, and the null hypothesis was rejected as normal distribution. Combined with Figure 1-A, it indicated that the gradation distribution of pakchoi canopy was skewed distribution.

| Model | | R-square | Adjusted R-square | RMSE | F value | Significance F |
| --- | --- | --- | --- | --- | --- | --- |
| *R*_Mean_ | Y=942.015+0.466 *RH*_h_ -0.813 *AP*_h_ -1.024 *TD*_h_ | 0.369 | 0.352 | 10.115 | 21.869 | 0.000 |
| *R*_Median_ | Y=1141.802-1.005 *AP*_h_ +0.469 *RH*_h_ -0.938 *TD*_h_ | 0.415 | 0.399 | 10.854 | 26.461 | 0.000 |
| *R*_Mode_ | Y=1428.922-1.261 *AP*_h_ +0.239 *RH*_h_ | 0.319 | 0.306 | 16.002 | 26.411 | 0.000 |
| *R*_Skewness_ | Y=-18.074+0.018 *AP*_h_ -0.005 *RH*_h_ | 0.513 | 0.504 | 0.178 | 59.419 | 0.000 |
| *R*_Kurtosis_ | Unable to modeling |  |  |  |  |  |
| *G*_Mean_ | Y=490.800+0.453 *RH*_h_ -0.353 *AP*_h_ | 0.512 | 0.503 | 8.677 | 59.194 | 0.000 |
| *G*_Median_ | Y=654.489+0.494 *RH*_h_ -0.512 *AP*_h_ | 0.535 | 0.527 | 9.619 | 65.109 | 0.000 |
| *G*_Mode_ | Y=2485.302-2.228 *AP*_h_ | 0.123 | 0.115 | 38.465 | 15.932 | 0.000 |
| *G*_Skewness_ | Y=-12.610-0.008 *RH*_h_ +0.012 *AP*_h_ | 0.498 | 0.489 | 0.189 | 55.964 | 0.000 |
| *G*_Kurtosis_ | Y=2.723+0.008 *RH*_h_ | 0.074 | 0.066 | 0.465 | 9.105 | 0.003 |
| *B*_Mean_ | Y=90.522+0.276 *RH*_h_ | 0.239 | 0.233 | 7.891 | 35.869 | 0.000 |
| *B*_Median_ | Y=86.626+0.346 *RH*_h_ | 0.289 | 0.282 | 8.715 | 46.258 | 0.000 |
| *B*_Mode_ | Y=124.269+2.031 *TD*_h_ | 0.323 | 0.317 | 13.504 | 54.441 | 0.000 |
| *B*_Skewness_ | Y=-8.071-0.009 *TD*_h_ +0.008P-0.004RH | 0.447 | 0.432 | 0.158 | 30.145 | 0.000 |
| *B*_Kurtosis_ | Unable to modeling |  |  |  |  |  |
| *Y*_Mean_ | Y=495.740+0.384 *RH*_h_ -0.364 *AP*_h_ | 0.454 | 0.445 | 8.603 | 47.042 | 0.000 |
| *Y*_Median_ | Y=675.950+0.419 *RH*_h_ -0.539 *AP*_h_ | 0.496 | 0.487 | 9.380 | 55.509 | 0.000 |
| *Y*_Mode_ | Y=1133.568+0.481 *RH*_h_ -0.983 *AP*_h_ | 0.437 | 0.427 | 14.370 | 43.855 | 0.000 |
| *Y*_Skewness_ | Y=-14.522-0.007 *RH*_h_ +0.014 *AP*_h_ | 0.512 | 0.504 | 0.179 | 59.328 | 0.000 |
| *Y*_Kurtosis_ | Unable to modeling |  |  |  |  |  |

**S2 Table .** Canopy color-meteorological response models based on the unclassified group (T0) of pakchoi and their goodness of fit (n=116)

|  | Models | R-square | Adjusted R-square | RMSE | F value | Significance F |
| --- | --- | --- | --- | --- | --- | --- |
| *R*_Mean_ | Y=1041.618-0.876 *AP*_h_ | 0.158 | 0.134 | 10.173 | 6.738 | 0.014 |
| *R*_Median_ | Y=1214.356-1.042 *AP*_h_ | 0.192 | 0.169 | 10.738 | 8.551 | 0.006 |
| *R*_Mode_ | Y=1513.492-1.326 *AP*_h_ | 0.167 | 0.143 | 14.901 | 7.194 | 0.011 |
| *R*_Skewness_ | Y=0.160-0.005 *RH*_h_ | 0.247 | 0.226 | 0.148 | 11.830 | 0.001 |
| *R*_Kurtosis_ | Unable to modeling |  |  |  |  |  |
| *G*_Mean_ | Y=132.454+0.408 *RH*_h_ | 0.335 | 0.316 | 8.982 | 18.100 | 0.000 |
| *G*_Median_ | Y=142.005+3.494 *VP*_h_ | 0.374 | 0.357 | 9.632 | 21.546 | 0.000 |
| *G*_Mode_ | Y=91.960+1.217 *RH*_h_ | 0.247 | 0.226 | 33.195 | 11.782 | 0.002 |
| *G*_Skewness_ | Y=-0.081-0.050 *VP*_h_ | 0.321 | 0.302 | 0.156 | 16.986 | 0.000 |
| *G*_Kurtosis_ | Unable to modeling |  |  |  |  |  |
| *B*_Mean_ | Y=97.955+1.699 *VP*_h_ | 0.201 | 0.179 | 7.218 | 9.073 | 0.005 |
| *B*_Median_ | Y=95.966+2.283 *VP*_h_ | 0.276 | 0.256 | 7.875 | 13.752 | 0.001 |
| *B*_Mode_ | Y=96.517+3.907 *VP*_h_ | 0.256 | 0.235 | 14.210 | 12.371 | 0.001 |
| *B*_Skewness_ | Y=0.328-0.050 *VP*_h_ | 0.367 | 0.349 | 0.140 | 20.833 | 0.000 |
| *B*_Kurtosis_ | Y=3.542-0.064*T*_h_ | 0.113 | 0.089 | 0.363 | 4.596 | 0.039 |
| *Y*_Mean_ | Y=125.832+0.333 *RH*_h_ | 0.265 | 0.244 | 8.652 | 12.965 | 0.001 |
| *Y*_Median_ | Y=133.459+2.913 *VP*_h_ | 0.307 | 0.288 | 9.326 | 15.973 | 0.000 |
| *Y*_Mode_ | Y=125.366+4.881 *VP*_h_ | 0.350 | 0.332 | 14.186 | 19.381 | 0.000 |
| *Y*_Skewness_ | Y=-0.011-0.047 *VP*_h_ | 0.316 | 0.297 | 0.147 | 16.608 | 0.000 |
| *Y*_Kurtosis_ | Unable to modeling |  |  |  |  |  |

**S3 Table .** Canopy color-meteorological response models based on the samples of the first type (T1) of pakchoi and their goodness of fit (n=38)

| Models | | R-square | Adjusted R-square | RMSE | F value | Significance F |
| --- | --- | --- | --- | --- | --- | --- |
| *R*_Mean_ | Y=821.538+3.936 *VP*_h_-0.680 *AP*_h_ | 0.450 | 0.435 | 10.043 | 30.661 | 0.000 |
| *R*_Median_ | Y=1065.848-0.917 *AP*_h_ +4.127 *VP*_h_ | 0.494 | 0.481 | 10.820 | 36.671 | 0.000 |
| *R*_Mode_ | Y=2170.385-1.961 *AP*_h_ | 0.359 | 0.350 | 16.561 | 42.536 | 0.000 |
| *R*_Skewness_ | Y=-21.535+0.021 *AP*_h_ -0.006 *RH*_h_ | 0.557 | 0.545 | 0.189 | 47.204 | 0.000 |
| *R*_Kurtosis_ | Unable to modeling |  |  |  |  |  |
| *G*_Mean_ | Y=124.649+6.961 *VP*_h_ | 0.557 | 0.551 | 8.678 | 95.509 | 0.000 |
| *G*_Median_ | Y=671.585+6.362 *VP*_h_ -0.525 *AP*_h_ | 0.589 | 0.579 | 9.608 | 53.847 | 0.000 |
| *G*_Mode_ | Y=2708.254-2.441 *AP*_h_ | 0.129 | 0.118 | 39.985 | 11.303 | 0.001 |
| *G*_Skewness_ | Y=-14.738-0.009 *RH*_h_ +0.015 *AP*_h_ | 0.546 | 0.534 | 0.198 | 45.097 | 0.000 |
| *G*_Kurtosis_ | Y=2.730+0.112 *VP*_h_ | 0.097 | 0.085 | 0.477 | 8.152 | 0.006 |
| *B*_Mean_ | Y=85.599+0.347 *RH*_h_ | 0.347 | 0.339 | 7.723 | 40.447 | 0.000 |
| *B*_Median_ | Y=86.809+4.853 *VP*_h_ | 0.385 | 0.377 | 8.574 | 47.558 | 0.000 |
| *B*_Mode_ | Y=126.396+2.625*TD*_h_ | 0.370 | 0.362 | 12.858 | 44.621 | 0.000 |
| *B*_Skewness_ | Y=-11.050-0.006 *RH*_h_ +0.011 *AP*_h_ | 0.475 | 0.461 | 0.160 | 33.864 | 0.000 |
| *B*_Kurtosis_ | Unable to modeling |  |  |  |  |  |
| *Y*_Mean_ | Y=116.275+6.339 *VP*_h_ | 0.513 | 0.507 | 8.628 | 80.144 | 0.000 |
| *Y*_Median_ | Y=710.198+5.569 *VP*_h_ -0.570 *AP*_h_ | 0.567 | 0.556 | 9.313 | 49.203 | 0.000 |
| *Y*_Mode_ | Y=1212.039+6.502 *VP*_h_ -1.057 *AP*_h_ | 0.537 | 0.525 | 13.568 | 43.556 | 0.000 |
| *Y*_Skewness_ | Y=-16.961-0.008 *RH*_h_+0.017 *VP*_h_ | 0.557 | 0.545 | 0.189 | 47.147 | 0.000 |
| *Y*_Kurtosis_ | Unable to modeling |  |  |  |  |  |

**S4 Table .** Canopy color-meteorological response models based on the samples of the second type (T2) of pakchoi and their goodness of fit (n=78)

| Models | | R-square | Adjusted R-square | RMSE | F value | Significance F |
| --- | --- | --- | --- | --- | --- | --- |
| *T*_h_ | Y=1.650+0.109 *B*_Mode_ -0.104 *B*_Mean_ | 0.225 | 0.212 | 2.388 | 16.440 | 0.000 |
| *RH*_h_ | Y=-54.504+1.609 *G*_Median_-0.308 *R*_Mode_-0.109*G*_Mode_-6.654*R*_Kurtosis_-0.395*B*_Mean_ | 0.610 | 0.592 | 10.215 | 34.396 | 0.000 |
| *AP*_h_ | Y=1034.582+11.796*R*_Skewness_+0.313*B*_Mean_-0.256*Y*_Median_ | 0.493 | 0.479 | 4.638 | 36.231 | 0.046 |
| *VP*_h_ | Y=-1.257-3.422 *B*_Skewness_ +0.046 *G*_Median_ | 0.391 | 0.381 | 1.641 | 36.338 | 0.000 |
| *TD*_h_ | Y=-34.347+0.206 *G*_Median_ | 0.399 | 0.393 | 3.563 | 75.569 | 0.000 |

**S5 Table .** Meteorological fitting models based on based on the unclassified group (T0) of pakchoi and their goodness of fit (n=116)

|  | Models | R-square | Adjusted R-square | RMSE | F value | Significance F |
| --- | --- | --- | --- | --- | --- | --- |
| *T*_h_ | Y=-5.810+0.108 *B*_Median_ | 0.242 | 0.221 | 1.774 | 11.521 | 0.002 |
| *RH*_h_ | Y=-21.266+1.471 *G*_Median_-1.296 *B*_Mean_ | 0.486 | 0.457 | 11.344 | 16.542 | 0.000 |
| *AP*_h_ | Y=1027.531+13.945*R*_Skewness_ | 0.224 | 0.203 | 4.423 | 10.407 | 0.003 |
| *VP*_h_ | Y=-9.883+0.107*G*_Median_ | 0.374 | 0.357 | 1.687 | 21.546 | 0.000 |
| *TD*_h_ | Y=-30.291+0.201*G*_Median_ | 0.351 | 0.333 | 3.330 | 19.434 | 0.000 |

**S6 Table .** Meteorological fitting models based on based on the samples of the first type (T1) of pakchoi and their goodness of fit (n=38)

| Models | | R-square | Adjusted R-square | RMSE | F value | Significance F |
| --- | --- | --- | --- | --- | --- | --- |
| *T*_h_ | Y=-2.680+0.039 *B*_Mode_ | 0.215 | 0.205 | 1.192 | 20.875 | 0.000 |
| *RH*_h_ | Y=-70.290+1.668 *G*_Median_ -0.420 *R*_Mode_-10.586 *R*_Kurtosis_-0.154 *G*_Mode_ | 0.744 | 0.730 | 8.399 | 52.954 | 0.000 |
| *AP*_h_ | Y=1032.128+15.627*R*_Skewness_ | 0.532 | 0.519 | 4.352 | 42.568 | 0.000 |
| *VP*_h_ | Y=-7.490+0.132*G*_Median_-0.014 *G*_Mode_-0.812 *Y*_Kurtosis_-0.023 *R*_Mode_ | 0.697 | 0.680 | 0.785 | 41.914 | 0.000 |
| *TD*_h_ | Y=-38.502+0.384 *G*_Median_-0.048 *G*_Mode_-2.063 *G*_Kurtosis_-0.059 *R*_Mode_ | 0.716 | 0.700 | 2.041 | 46.003 | 0.000 |

**S7 Table .** Meteorological fitting models based on based on the samples of the second type (T2) of pakchoi and their goodness of fit (n=78)
